# Low Growth Hormone Levels in Short-Stature Children with Pituitary Hyperplasia Secondary to Primary Hypothyroidism

**DOI:** 10.1155/2015/283492

**Published:** 2015-09-02

**Authors:** Minghua Liu, Yanyan Hu, Guimei Li, Wenwen Hu

**Affiliations:** ^1^Department of Pediatrics, Shandong Provincial Hospital Affiliated to Shandong University, 9677 Jingshi Road, Jinan, Shandong 250014, China; ^2^Department of Child Health Care, Shandong Maternal and Child Health Care Hospital, 238 Jingshi East Road, Jinan, Shandong 250014, China; ^3^Department of Pediatrics, The People's Hospital of Lanshan District, 51 Lanshan Road, Linyi, Shandong 276000, China

## Abstract

*Objective.* The follow-up of GH levels in short-stature children with pituitary hyperplasia secondary to primary hypothyroidism (PPH) is reported in a few cases. We aimed to observe changes in GH secretion in short-stature children with PPH. *Methods.* A total of 11 short-stature children with PPH accompanied by low GH levels were included. They received levothyroxine therapy after diagnosis. Their thyroid hormones, IGF-1, PRL, and pituitary height were measured at baseline and 3 months after therapy. GH stimulation tests were performed at baseline and after regression of thyroid hormones and pituitary. *Results.* At baseline, they had decreased GH peak and FT_3_ and FT_4_ levels and elevated TSH levels. Decreased IGF-1 levels were found in seven children. Elevated PRL levels and positive thyroid antibodies were found in 10 children. The mean pituitary height was 14.3 ± 3.8 mm. After 3 months, FT_3_, FT_4_, and IGF-1 levels were significantly increased (all *p* < 0.01), and values of TSH, PRL, and pituitary height were significantly decreased (all *p* < 0.001). After 6 months, pituitary hyperplasia completely regressed. GH levels returned to normal in nine children and were still low in two children. *Conclusion.* GH secretion can be resolved in most short-stature children with PPH.

## 1. Introduction

Primary hypothyroidism is a common endocrine disorder in children. Pituitary hyperplasia secondary to primary hypothyroidism (PPH) has been described in case reports [1–3]. Khawaja et al. [4] reported that pituitary hyperplasia was present in 90% of primary hypothyroidism children with serum thyrotropin (TSH) ≥ 50 mIU/L. Children with PPH rarely present with neurological symptoms [5]. The predominant clinical manifestation is short stature in most of them. Primary hypothyroidism is also a well-recognized etiology of low growth hormone (GH) levels [6]. Regression of the pituitary hyperplasia and low GH levels after adequate thyroid hormone replacement therapy have been reported separately [2, 7]. To our knowledge, only a few cases of children with PPH accompanied by low GH levels have been reported in the literature [8]. Few studies have investigated changes in GH secretion in short-stature children with PPH. To clarify this issue, we analyzed clinical, hormonal, and imaging changes in a cohort of short-stature children with PPH accompanied by low GH levels before and after 3 months of thyroid hormone replacement therapy.

## 2. Subjects and Methods

### 2.1. Study Subjects

This retrospective study was carried out at the Department of Pediatrics of Shandong Provincial Hospital, Shandong University, between March 2010 and October 2013. The study was approved by the ethical committee of the hospital. Written informed consent was obtained from parents.

We encountered 13 children with PPH whose chief complaint was short stature. Short stature was defined as height of children less than the 3rd percentile. The diagnosis of PPH was based on typical symptoms and signs of hypothyroidism, low free thyroxine (FT_4_) level with high TSH level, and regression of the hyperplastic pituitary after thyroid hormone replacement therapy. We excluded children with sexual precocity or history of other endocrine disorders or systemic diseases. Children who had taken thyroid hormones and/or recombinant human growth hormone (rhGH) before the study were also excluded. 11 children with low GH levels (GH peak ≤ 10 ng/mL) were included in the final analysis.

The following parameters were collected at baseline: chronological age (CA), bone age (BA), height, weight, pituitary height, and serum levels of GH peak, free triiodothyronine (FT_3_), FT_4_, TSH, thyroid peroxidase antibody (TPOAb), thyroglobulin antibody (TgAb), insulin-like growth factor-1 (IGF-1), and prolactin (PRL). All children were treated with levothyroxine (L-T_4_) (60–100 *μ*g/m^2^/d) after diagnosis. Thyroid function was examined every two weeks initially. Clinical, imaging, and biochemical abnormalities were evaluated after 3 months of therapy. Pituitary magnetic resonance imaging (MRI) was monitored until the pituitary resolved. GH stimulation tests were performed after regression of both thyroid hormones and pituitary height.

### 2.2. Assays

Height was measured to the nearest 0.1 cm by a standard stadiometer with the subjects wearing no shoes. Weight was measured to the nearest 0.1 kg by an electronic scale with the subjects wearing light clothing. Height was expressed as height standard deviation scores (HtSDS) using normative values for Chinese children [9]. Body mass index (BMI) was calculated as weight (kg)/height (m)^2^. Breast stages (B1–B5), genital stages (G1–G5), and pubic hair stages (PH1–PH5) were evaluated according to Tanner criteria [10]. Girls with stages of B1 (no breast development) and PH1 (no pubic hair) and boys with stages of G1 (testicular volume ≤ 3 mL) and PH1 were considered prepubertal. Clinical examinations were performed by the same investigator.

Fasting blood samples were obtained from subjects. The samples were clotted and centrifuged to obtain serum. Serum FT_3_, FT_4_, TSH, TPOAb, and TgAb levels were measured using chemiluminescence assay (ADVIA Centaur, Siemens Healthcare Diagnostics, USA). The intra- and interassay coefficients of variation (CVs) for thyroid hormones were <4.0% and <3.0%, respectively. Reference ranges for FT_3_, FT_4_, TSH, TPOAb, and TgAb were 3.5–6.5 pmol/L, 11.5–22.7 pmol/L, 0.35–5.5 mIU/L, 0–60 IU/mL, and 0–60 IU/mL, respectively. The sensitivity of FT_3_, FT_4_, TSH, TPOAb, and TgAb was 0.3 pmol/L, 1.3 pmol/L, 0.001 mIU/L, 0.1 IU/mL, and 10 IU/mL, respectively. Serum IGF-1 levels were measured using chemiluminescence assay (IMMULITE 2000, Siemens Healthcare Diagnostics, USA). The intra- and interassay CVs for the assays were 5.0% and 4.0%, respectively. Reference range for IGF-1 was different in different age. The sensitivity was 20 ng/mL. Serum GH and PRL levels were measured using chemiluminescence assay (Cobas E170, Roche Diagnostics, Germany). The intra- and interassay CVs for the assays were <5.0% and <8.0%, respectively. The reference range of PRL was 4.04–15.2 *μ*g/L for boys and 4.79–23.3 *μ*g/L for girls. The sensitivity of GH and PRL was 0.1 ng/mL and 0.047 *μ*g/L, respectively. Serum GH levels were determined at 0, 30, 60, 90, 120, and 150 min after two stimulation tests (arginine test and levodopa test). GH peak > 10 ng/mL should be considered normal.

BA was determined by radiograph of the left hand and wrist according to the method of Greulich and Pyle [11]. Ultrasound examination of the thyroid was performed on a LOGIQ 9 scanner (GE Medical Systems, Milwaukee, WI, USA) with a 5–12 MHz transducer. MRI scans of the pituitary were obtained in the sagittal and coronal planes on T1- and T2-weighted images using a 3.0 T scanner (Siemens, Erlangen, Germany), with 3 mm slice thickness. Contrast-enhanced MRI scans of the hyperplastic pituitary were performed. The height of the pituitary gland was recorded.

### 2.3. Statistical Analysis

Statistical analyses were conducted using SPSS version 17.0 program (Chicago, IL, USA). Parametric variables were expressed as the mean ± standard deviation. Nonparametric variables were expressed as median (interquartile range). Paired *t*-test (parametric test) was used to determine the significance of clinical, hormonal, and imaging changes before and after therapy. A two-tailed *p* value < 0.05 was considered statistically significant.

## 3. Results

### 3.1. Baseline Characteristics

Baseline characteristics of the 11 children (8 boys and 3 girls) are displayed in Table 1. Their average age was 10.3 ± 3.3 (range 5.0–15.8) years. Patients 7 (Tanner 2), 8 (Tanner 4), and 11 (Tanner 2) were in puberty. The remaining children were in prepuberty. The age at onset of hypothyroidism was 4.1 ± 3.5 years. The duration was 6.2 ± 3.6 years. All children showed typical symptoms and signs of hypothyroidism. Growth retardation was the most common manifestation (*n* = 11, 100%), followed by dry skin (*n* = 9, 81.8%; 6 boys and 3 girls). Other symptoms and signs were fatigue, sluggish speech, and slow movement (*n* = 7, 63.6%; 5 boys and 2 girls), weight gain (*n* = 6, 54.5%; 4 boys and 2 girls), puffy face and mental retardation (*n* = 5, 45.5%; 4 boys and 1 girl), and anemia (*n* = 4, 36.4%; 3 boys and 1 girl). Liver dysfunction was suggested by elevated aminotransferases (*n* = 8, 72.7%; 7 boys and 1 girl); five of the seven boys had elevated cholesterol and low-density lipoprotein (LDL) cholesterol levels. Visual symptoms and signs were not found in all children.

As shown in Table 1, all children had decreased GH peak and FT_3_ and FT_4_ levels, elevated TSH levels, and delayed BA. Their BA was 6.9 ± 3.4 (range 1.0–13.0) years. Serum IGF-1 levels were decreased in seven (63.6%) children and normal in four children (patients 1, 6, 9, and 11). Serum PRL levels were elevated in 10 (90.9%) children and normal in one child (patient 3). TPOAb and/or TgAb were positive in 10 (90.9%) children. Their thyroid ultrasound showed enlarged thyroid glands with heterogeneous echo texture and hypoechogenicity. They were diagnosed with Hashimoto's thyroiditis (HT). The thyroid ultrasound in the remaining one patient was normal. MRI scans showed homogeneously enlarged pituitary gland with suprasellar extension in 11 children (100%). Of the 11 children, two children (18.2%) had increased thickness of pituitary stalk, two children (18.2%) had invisible pituitary stalk, 3 children (27.3%) had compressed optic chiasm, and one child (9.1%) had Rathke's cleft cyst.

### 3.2. After L-T_4_ Treatment

**Figure 1:**
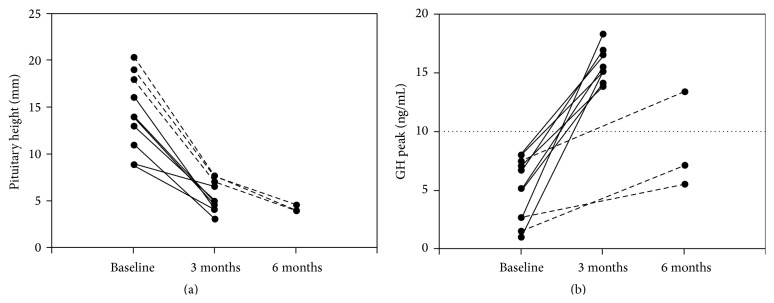
Changes in pituitary hyperplasia and low GH levels in short-stature children with PPH before and after L-T_4_ therapy. In eight children (solid lines), pituitary hyperplasia and low GH levels returned to normal after 3 months of L-T_4_ therapy. In three children (patients 4, 5, and 6) (dotted lines), pituitary hyperplasia returned to normal after 6 months of L-T_4_ therapy. GH levels returned to normal in patient 4 and were still low in patients 5 and 6.

The clinical symptoms and signs of hypothyroidism improved after 3 months of L-T_4_ treatment. Thyroid function tests returned to normal. HtSDS values were significantly higher than before treatment (*p* = 0.006). BMI values were significantly lower than before treatment (*p* = 0.004). Abnormalities in liver enzymes, blood lipids, and blood routine examinations were corrected. Serum FT_3_, FT_4_, and IGF-1 levels were significantly higher than before treatment (*p* < 0.001, *p* < 0.001, and *p* = 0.004). Serum TSH and PRL levels were significantly lower than before treatment (*p* < 0.001, *p* < 0.001). The pituitary height was significantly less than before treatment (*p* < 0.001) (Table 2). MRI scans showed complete resolution of the hyperplastic pituitary in eight children (patients 1, 2, 3, 7, 8, 9, 10, and 11). In three children (patients 4, 5, and 6), the pituitary height after 3 months of therapy decreased by 7 mm, 7.5 mm, and 7.7 mm, respectively. Reassessment of pituitary MRI scans after 6 months of therapy revealed normal size of pituitary in those three children. Two children (patients 5 and 6) still had subnormal GH peak levels (5.5 ng/mL and 7.1 ng/mL, resp.) despite resolution of both thyroid function and pituitary height. In other children, repeat GH peak levels returned to normal and were more than 10 ng/mL. GH peak levels increased from 5.0 ± 2.7 ng/mL to 13.8 ± 4.0 ng/mL after L-T_4_ therapy (Figure 1).

## 4. Discussion

In the present study of short-stature children with PPH accompanied by low GH levels, decreased FT_3_, FT_4_, IGF-1, and GH levels and elevated values of TSH, PRL, and pituitary height were observed. Low growth hormone levels could be resolved after thyroid hormone replacement therapy in most short-stature children with PPH. Thyroid hormone replacement therapy led to improvement of thyroid hormones, IGF-1, GH and PRL levels, and the enlarged pituitary. Primary hypothyroidism is the main cause of these hormonal and imaging changes.

The association of pituitary hyperplasia and primary hypothyroidism was first reported by Niepce in 1851 [12]. Sporadic cases of pituitary hyperplasia in patients with primary hypothyroidism have been published since then. PPH has been well described. In long-standing primary hypothyroidism, insufficient production of thyroid hormones directly or indirectly leads to overproduction of TSH and thyrotropin-releasing hormone (TRH) due to the loss of thyroxine feedback inhibition [13, 14]. Elevated TRH levels stimulate both pituitary thyrotrophs and lactotrophs hypertrophy and subsequent enlargement of the pituitary gland, resulting in oversecretion of TSH and PRL levels. Histological examination of the enlarged pituitary gland in patients with primary hypothyroidism has shown hyperplasia of both thyrotrophs and lactotrophs [15, 16]. Inhibition of hypothalamic dopamine in hypothyroidism could also increase PRL secretion [17]. The reported incidence of hyperprolactinemia was 75% in patients with PPH [18]. In our study, 90.9% of short-stature children with PPH accompanied by low GH levels had elevated PRL levels. Only one boy had normal PRL levels. It probably resulted from compression of infundibulum by the enlarged pituitary [19].

The normal pituitary gland undergoes dramatic changes in size and shape due to physiological alterations in hormones during puberty. Pituitary enlargement is much more prominent in girls in puberty, but it can also occur in boys. The pituitary gland can reach a height of 8–10 mm in girls and 7 mm in boys [20]. The pituitary hyperplasia during puberty can resolve after puberty. However, hyperplastic pituitary glands in our prepubertal and pubertal children were more dramatic. Regression in pituitary hyperplasia after L-T_4_ therapy on follow-up MRI scans can be distinguished from pituitary adenoma.

GH secretion could be decreased in short-stature children with PPH and accompanied by delayed BA. In our small number of short-stature children with PPH, we found that 84.6% of them had low GH levels. All 11 children showed delayed BA. Upward convexity of the pituitary gland results in thick or invisible pituitary stalk on MRI. Compression of the pituitary stalk and infundibulum in patients with PPH might contribute to the influence on GH secretion. Compression of the somatotrophs by hyperplastic thyrotrophs and lactotrophs could decrease GH secretion. Transformation of somatotrophs to thyrotrophs in the pituitary of patients with primary hypothyroidism might also reduce GH secretion [21]. Moreover, thyroid hormones are essential for growth, development, and metabolism. Thyroxine is a stimulating factor for GH synthesis. Reduced GH secretion and decreased activity of thyroid hormones in the synthesis of proteins cause short stature and delayed BA [7]. Serum IGF-1 level is influenced by multiple factors, such as age, gender, puberty, nutrition, thyroid status, and prolactin status [22, 23]. The reference range for IGF-1 is different. Therefore, IGF-1 level might be normal in our four children. Thyroid hormones regulate basal metabolic rate of hepatocytes, increase degradation of blood lipids, and stimulate erythropoiesis. Elevated liver enzymes and blood lipids and anemia subsequently develop in hypothyroid state.

HT is considered to be the most common cause of primary hypothyroidism in children [24]. A positive correlation between pituitary size and TSH levels has been previously reported [4]. In this study, most of our children had HT. Our children had a long duration of symptoms probably due to early unconspicuous symptoms and lack of clinical awareness about the disease and neonatal screening of congenital hypothyroidism in remote rural areas. As a result, remarkably high TSH levels followed by pituitary hyperplasia were found in children with primary hypothyroidism.

After thyroid hormone replacement therapy, clinical symptoms improved, liver function, blood lipids, and blood routine examinations returned to normal, thyroid hormones and PRL levels resolved to the normal range, and MRI scans showed regression in the size of the enlarged pituitary. Although GH secretion increased in all short-stature children with PPH after regression in hormonal levels and pituitary size, there were two boys in our study whose GH secretion remained abnormal. An intrinsic defect in GH secretion should be considered. Based on the resolution in clinical, hormonal, and imaging changes after thyroid hormone replacement therapy, the diagnosis of primary hypothyroidism was definite. The time interval varied from one week to 12 months in different cases [5, 25]. In our children, all abnormalities resolved after 6 months of therapy.

GH secretion can be resolved in most short-stature children with PPH. Primary hypothyroidism is associated with low GH levels and reversible pituitary hyperplasia in addition to abnormal thyroid function and PRL levels. The recognition of these associations between primary hypothyroidism and pituitary size as well as GH secretion can avoid misdiagnosis and eliminate unnecessary harmful therapy. Additional studies with larger samples are needed to clarify more the associations between primary hypothyroidism and hormonal and imaging abnormalities. Animal studies are required to investigate the mechanism.

## Figures and Tables

**Table 1:** Baseline characteristics of short-stature children with PPH accompanied by low GH levels.

Patient	CA (years)	BA (years)	GH peak (ng/mL)	FT_3_ (pmol/L)	FT_4_ (pmol/L)	TSH (mIU/L)	TPOAb (IU/mL)	TgAb (IU/mL)	IGF-1 (ng/mL)	PRL (*μ*g/L)	Pituitary height (mm)
1 M	6.2	1.0	8.0	1.6	3.4	>150	42.1	23.9	145.0	49.1	11.0
2 M	9.8	6.0	2.7	0.1	3.0	>150	>500	>1300	78.0	32.2	14.0
3 M	11.2	8.0	5.1	1.2	4.3	>150	>500	>1300	97.7	12.5	16.0
4 M	10.7	6.0	7.5	2.0	3.3	>150	73.8	>1300	68.61	52.6	18.0
5 M	10.8	7.0	2.6	2.6	4.3	75.0	>500	>1300	56.7	27.5	20.3
6 M	7.9	4.0	1.5	3.0	6.6	>150	>500	404.5	106.98	52.4	19.0
7 M	15.0	13.0	6.7	4.9	6.7	65.5	>500	>1300	147.42	28.5	14.0
8 M	15.8	10.0	8.0	1.0	1.2	>150	627.3	36.9	148.3	41.5	14.0
9 F	5.0	3.0	7.1	0.2	3.6	>150	>500	>1300	75.26	75.4	13.0
10 F	9.3	8.5	5.2	6.5	7.3	78.0	>500	600.9	63.0	33.2	8.8
11 F	11.5	9.0	1.0	0.8	1.4	>150	>500	>1300	123.0	41.4	9.0

PPH: pituitary hyperplasia secondary to primary hypothyroidism; GH: growth hormone; M: male; F: female; CA: chronological age; BA: bone age; FT_3_: free triiodothyronine; FT_4_: free thyroxine; TSH: thyrotropin; TPOAb: thyroid peroxidase antibody; TgAb: thyroglobulin antibody; IGF-1: insulin-like growth factor-1; PRL: prolactin.

**Table 2:** Parameters in short-stature children with PPH accompanied by low GH levels before and after 3 months of L-T_4_ therapy.

Parameters	Before L-T_4_ therapy (*n* = 11)	After L-T_4_ therapy (*n* = 11)	*p* value
HtSDS	−2.96 ± 0.73	−2.51 ± 0.96	0.006
BMI	22.14 ± 5.91	19.09 ± 4.46	0.004
FT_3_ (pmol/L)	2.2 ± 2.0	7.6 ± 1.8	<0.001
FT_4_ (pmol/L)	4.1 ± 2.1	20.4 ± 4.5	<0.001
TSH (mIU/L)	>50.0	1.9 ± 2.0	<0.001
IGF-1 (ng/mL)	100.91 ± 35.36	129.74 ± 46.07	0.004
PRL (*μ*g/L)	40.6 ± 16.7	8.7 ± 3.1	<0.001
Pituitary height (mm)	14.3 ± 3.8	5.4 ± 1.5	<0.001

PPH: pituitary hyperplasia secondary to primary hypothyroidism; GH: growth hormone; L-T_4_: levothyroxine; HtSDS: height standard deviation scores; BMI: body mass index; FT_3_: free triiodothyronine; FT_4_: free thyroxine; TSH: thyrotropin; IGF-1: insulin-like growth factor-1; PRL: prolactin.
